# Baseline Resting-State Network Integration Modulates Task Performance and Aftereffect

**DOI:** 10.3390/s26010041

**Published:** 2025-12-20

**Authors:** Rok Požar, Tim Martin, Mary Katherine Kerlin, Aidan McColligan, Bruno Giordani, Voyko Kavcic

**Affiliations:** 1 Faculty of Mathematics, Natural Sciences and Information Technologies, University of Primorska, Glagoljaška 8, 6000 Koper, Slovenia; 2 Andrej Marušič Institute, University of Primorska, 6000 Koper, Slovenia; 3 Institute of Mathematics, Physics and Mechanics, 1000 Ljubljana, Slovenia; 4 Kennesaw State University, Kennesaw, GA 30144, USA; tmarti61@kennesaw.edu (T.M.); mkerlin2@students.kennesaw.edu (M.K.K.); amccol24@students.kennesaw.edu (A.M.); 5 Michigan Alzheimer’s Disease Research Center, Ann Arbor, MI 48109, USA; giordani@med.umich.edu; 6 Department of Psychiatry, University of Michigan, Ann Arbor, MI 48109, USA; 7 Institute of Gerontology, Wayne State University, Detroit, MI 48202, USA; voyko@wayne.edu; 8 International Institute of Applied Gerontology, 1000 Ljubljana, Slovenia

**Keywords:** electroencephalography, brain network integration, cognitive task, neuropsychology

## Abstract

Understanding how intrinsic brain networks adapt to cognitive demands is central to neuroscience. The aim of this study was to examine how eyes-open and eyes-closed resting-state network integration, derived from electroencephalography before and after a visual oddball task, relates to task performance in young adults. Task engagement reduced global integration in theta, lower alpha, and beta bands, independent of eye condition, indicating a transient shift toward a less demanding post-task configuration. Eyes-open resting states consistently exhibited higher integration than eyes-closed in the upper alpha band, both before and after the task, reflecting enhanced inter-regional communication and sensory readiness. Importantly, higher pre-task beta-band integration during eyes-open resting state predicted faster reaction times and larger post-task decreases in integration, highlighting baseline network organization as a determinant of cognitive efficiency and neural flexibility. These findings support the concept of neural reserve, where intrinsic network efficiency and adaptability underpin both performance readiness and dynamic reorganization. Overall, the results demonstrate that resting-state network integration—modulated by both eye condition and task engagement—captures fundamental aspects of the brain’s capacity for efficient and flexible cognitive function.

## 1. Introduction

Understanding how intrinsic brain networks support cognitive performance and adapt to task demands is central to cognitive neuroscience. Cognition arises from dynamic interactions among distributed brain regions, reflecting the brain’s deeply interconnected network organization [1]. Integration—a hallmark of this organization—allows for specialized, spatially distant regions to coordinate, combining their distinct contributions into a unified system [2]. This dynamic process enables the brain to flexibly adapt, supporting complex and shifting cognitive demands [3,4,5,6,7,8,9,10,11,12].

Even at rest, the brain remains highly active [13], reflecting its intrinsic capacity for information processing [14]. Resting-state network integration appears to capture this latent potential, with higher integration linked to faster cognitive control [15], better prospective memory [16], greater intelligence [17], and enhanced cognitive ability [18]. Yet resting networks are not static: task engagement can leave measurable aftereffects [19,20]. In young adults, attentional and visual tasks reduce network integration from pre- to post-task resting state [21,22], whereas in older adults, integration may increase following a motion direction discrimination task [23]. Whether such aftereffects reflect stable network traits that promote efficiency and flexibility or transient adjustments tied to task strategies remains unclear.

Resting-state dynamics are further influenced by eye condition [24]. Both increases and decreases in global network integration have been reported when comparing eyes-open and eyes-closed resting states [25,26,27,28]. Eye condition thus provides a critical context for interpreting task-induced changes in network organization.

Here, we extend previous studies by jointly examining how task engagement, performance, and eye condition shape resting-state network integration. Using electroencephalography (EEG), we recorded eyes-closed and eyes-open resting states immediately before and after an oddball paradigm in healthy young adults. This design allowed for the assessment of not only whether task engagement alters network integration, but also whether baseline or post-task integration and the magnitude of change predict performance, as well as how these effects interact with eye condition—a factor often considered in isolation. Network structure was reconstructed using maximum spanning trees as the backbone [29], and overall network integration was derived from the resulting topology.

## 2. Materials and Methods

### 2.1. Participants

Forty-five undergraduate students participated in the partial fulfillment of a course requirement at Kennesaw State University in the United States. Nine participants were excluded from the current sample because post-task resting state measurements could not be taken within the allotted time. Behavioral data from two additional participants were lost because of a faulty stimulus cable, although their data were retained in the comparison of pre-task and post-task spectral power. Of the thirty-six participants with both pre-task and post-task rsEEG, 22 (61%) were female, 32 (88.9%) reported being right-handed, all reported normal or corrected visual acuity, and one (2.8%) reported being red–green colorblind. There were 8 (22.2%) Hispanic, 8 (22.2%) Black or African American, 5 (13.9%) Asian American or Pacific Islander, 1 (2.8%) American Indian or Alaska Native, and 14 (38.9%) White, Non-Hispanic participants. The mean age was 19.28 s ±1.61 years.

### 2.2. Study Design

The experimental design was identical to that reported previously [20]. Briefly, participants completed resting-state EEG recordings both immediately before and immediately after the oddball task. Each resting-state session consisted of two alternating 1 min eyes-closed and eyes-open periods. During the task, participants performed a standard oddball go/no-go task with geometric shapes. Each block (36 total) contained 30 stimuli: one target (10%), one rare foil (10%), and one common foil (80%). Participants responded to targets with a button press. Stimulus presentation and counterbalancing of target assignment followed the procedure described in Martin et al. [20].

### 2.3. Behavioral Data Analysis

Behavioral data were available for thirty-four participants. Behavioral measures included reaction time, hit rate, and false alarm rate. Perceptual sensitivity, independent of response bias, was quantified using the signal detection measure *d′*, calculated from hit and false alarm rates according to signal detection theory [30]. In four cases where no false alarms were emitted, a false alarm rate of 0.001 was substituted to allow for the calculation of *d′*.

### 2.4. EEG Recording and Processing

EEG data preprocessing and cleaning have been described in detail previously [20]. Briefly, EEG data were recorded from a 64-channel SynAmps 2/Neuvo amplifier (Compumedics Neuroscan) using a Quik-Cap Neo Net (62 scalp electrodes), with impedances kept below 10 kΩ. Data were referenced to a site posterior to Cz, grounded anterior to Fz, and sampled at 1 kHz. Vertical and horizontal eye movements were monitored using electrodes above/below the left eye and at the left/right temples.

Offline preprocessing was performed in EEGLAB v2024.2 [31]. Data were downsampled to 500 Hz, re-referenced to linked earlobes, and band-pass filtered (0.03–45 Hz). Channels and epochs with high-amplitude noise were removed, and bad channels were interpolated. Blink and movement artifacts were identified and removed using independent component analysis (extended infomax; [32]).

After preprocessing, the continuous data were segmented into 2 s resting-state epochs for subsequent network integration analysis. For each subject and condition, the first 30 epochs were used to calculate functional connectivity across the following frequency bands: delta (0.5–4 Hz), theta (4–8 Hz), lower alpha (8–10 Hz), upper alpha (10–13 Hz), and beta (13–20 Hz). Functional connectivity between each pair of channels was quantified for each epoch using the phase lag index [33] and stored in a 62 × 62 connectivity matrix.

### 2.5. Network Construction

Functional connectivity matrices represented the brain as weighted graphs, where vertices corresponded to electrodes and edges encoded the strength of their functional interactions. To extract the backbone of these graphs, Kruskal’s algorithm [34] was applied to each matrix to compute a maximum spanning tree (MST). The MST is a cycle-free subgraph that preserves global connectivity while maximizing overall edge weight. Each tree consisted of 62 vertices and 61 edges, and edge weights were omitted from subsequent analyses. This procedure enforced uniform network density across participants, ensuring that observed differences reflected variations in topological organization rather than disparities in connection strength or density.

### 2.6. Network Integration

To assess network integration, we first calculated leaf number and pairwise distance. A leaf in a tree is a node connected by an edge to exactly one other node, and a leaf number corresponds to the total number of such nodes in the tree. The distance between two nodes is the number of edges on the (unique) path between them, with the largest distance defining the tree diameter. The eccentricity of a node in a tree is the greatest distance from that node to any other node in the tree. All measures were normalized to their theoretical maxima, and then leaf number, diameter, and mean eccentricity across all nodes were used as global indices in subsequent statistical analyses. We used the NetworkX library [35] (version 3.3) in Python 3.12.4 to compute MSTs and the corresponding topological measures.

Tree topology can range between two extremes. A star-like tree, with a single central hub connecting to all other nodes, maximizes integration by exhibiting high leaf number but minimal diameter and mean eccentricity. In contrast, a path-like tree, where nodes are connected sequentially, minimizes integration with low leaf number but maximal diameter and mean eccentricity. Thus, greater network integration is characterized by higher leaf number combined with shorter diameter and mean eccentricity.

### 2.7. Statistical Analysis

All statistical analyses were conducted in MATLAB R2025b (The MathWorks, Inc.). For each subject, MST measures were averaged across epochs before analysis. Outliers were identified within each frequency band using the interquartile range and excluded. Participants were removed if classified as outliers in any of the following conditions: pre-task eyes-closed, pre-task eyes-open, post-task eyes-closed, or post-task eyes-open resting states. Final sample sizes are reported in Table S1.

Changes in MST measures were tested using linear mixed-effects models fitted separately for each frequency band. Fixed factors included timepoint (pre-task and post-task coded as −1 and 1), eye state (closed and open coded as −1 and 1), and their interaction. Random intercepts for timepoints were included per subject. Multiple comparisons across frequency bands were controlled using the false discovery rate (FDR) procedure [36] with FDR-adjusted p <0.05 considered statistically significant.

To investigate the relationship between task performance and global integration, correlation analyses between reaction time and MST measures were conducted using Pearson’s correlations for participants included in the mixed models and with complete task performance data. Final sample sizes are reported in Table S4. Analyses were performed separately for pre- and post-task, and for eyes-closed and eyes-open conditions. To examine whether task-related change predicted reaction time independently of baseline, post-task values were regressed on pre-task values, and the residuals were correlated with reaction time. Correlation analyses were restricted to frequency bands showing significant timepoint effects in the mixed models.

## 3. Results

### 3.1. Behavioral Performance

The mean reaction time across participants was 0.45 ±0.03 s, with a mean hit rate of 0.78 ±0.16, a mean false alarm rate of 0.007 ±0.01, and a mean *d′* of 3.60 ±0.78.

### 3.2. Changes in Network Organization

**Figure 1:**
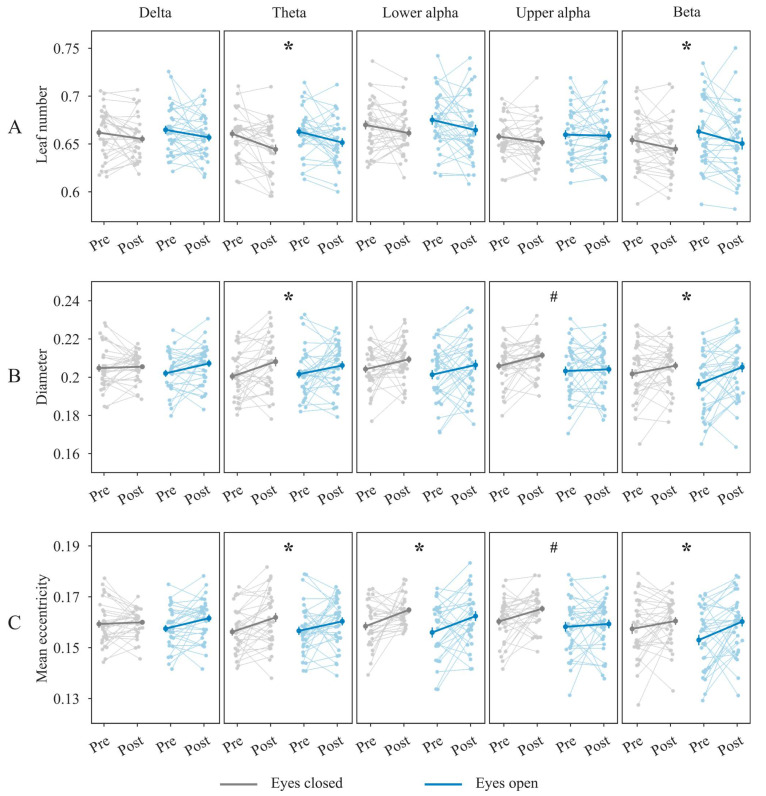
Leaf number (**A**), diameter (**B**), and mean eccentricity (**C**) from pre- to post-task resting-states across frequency bands and eye conditions. Each dot represents a participant’s value at a given time point (Pre, Post) and eye condition (gray—eyes-closed; blue—eyes-open). Bold dots indicate mean values, with error bars representing standard error. Mean values are connected by lines. Asterisk (*) indicates a significant main effect of time, and hashtag (#) indicates a significant main effect of eye status. FDR-corrected p <0.05 was considered significant.

We found that leaf number decreased from the pre-task to post-task timepoint across both eyes-closed and eyes-open conditions in the theta and beta bands (main effect of time: $p_{FDR}=\;0.002$ and $p_{FDR}=\;0.01$, respectively; see Figure 1A). In contrast, the diameter increased from the pre- to post-task timepoint in the theta and beta bands (main effect of time: $p_{FDR}=\;0.01$ and $p_{FDR}=\;0.006$, respectively; see Figure 1B). Similarly, mean eccentricity increased from the pre-task to post-task timepoint in the theta, lower alpha, and beta bands (main effect of time: $p_{FDR}=\;0.006$, $p_{FDR}=\;0.002$, and $p_{FDR}=\;0.005$, respectively; see Figure 1C). Together, these results suggest that post-task networks were less integrated compared to the pre-task networks in the theta, lower alpha, and beta bands.

Diameter and mean eccentricity were lower during eyes-open compared to eyes-closed conditions across both timepoints in the upper alpha band (main effect of eye status: $p_{FDR}=\;0.03$ and $p_{FDR}=\;0.02$, respectively; see Figure 1B,C). These findings indicate a more integrated network topology during eyes-open compared to eyes-closed conditions in the upper alpha band.

No other main effects or interactions reached statistical significance across any frequency band after correction for multiple comparisons across frequency bands (for full statistical details, see Table S1).

To further explore whether the magnitude of post-task reduction in network integration depended on baseline levels, we conducted an additional exploratory analysis in the theta, lower alpha, and beta bands, separately for eyes-closed and eyes-open conditions. We first correlated pre-task values with the corresponding (post − pre) change scores. Across all three frequency bands, both conditions, and all three MST measures, Pearson correlations were significantly negative, suggesting that the higher baseline integration was associated with larger decreases (full statistical details in Table S2). Because such correlations can be influenced by mathematical coupling, we then applied a more robust approach by fitting regression models of post-task values on pre-task values and testing whether slopes significantly differed from one, i.e., whether pre-task levels predicted change. Consistently across theta, lower alpha, and beta bands, for both eyes-closed and eyes-open conditions and all three MST measures, the estimated slopes were significantly below one (full statistical details in Table S3), indicating that higher pre-task integration predicted greater reductions.

### 3.3. Brain-Behavior Relationships

**Figure 2:**
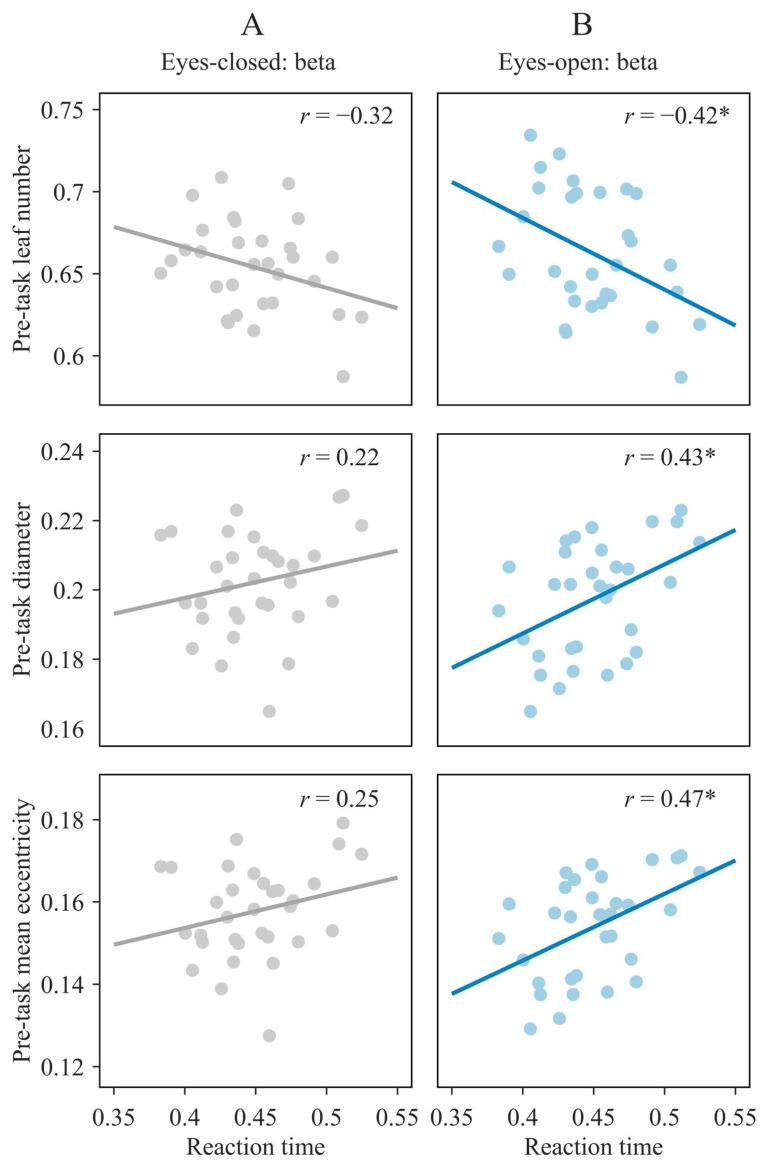
Brain–behavior relationships. Scatterplots show the correlations between reaction time and pre-task MST measures (leaf number, diameter, and mean eccentricity) in the beta frequency band for the eyes-closed (**A**) and eyes-open (**B**) conditions. Asterisk (*) denotes significant correlations (FDR-corrected p < 0.05); r = correlation coefficient.

In the beta band, faster reaction times were significantly associated with higher pre-task leaf number and lower pre-task diameter and mean eccentricity during the eyes-open condition (p = 0.02, p = 0.01 and p = 0.007, respectively; Figure 2B), but not during the eyes-closed condition (all p > 0.08; Figure 2A), indicating that higher pre-task network integration in the beta band during the eyes-open resting state is linked to faster responses.

To rule out potential effects of task sensitivity, three separate multiple regression analyses were performed with reaction time as the outcome and d′ as a covariate. Each model included one pre-task network measure (leaf number, diameter, or mean eccentricity) from the eyes-open condition. In all models, the MST measure remained significant (all p < 0.03), whereas d′ was not (all p > 0.31), confirming that these associations reflect performance efficiency beyond individual differences in sensitivity.

No significant correlations were found between reaction time and any pre-task MST measure in the lower alpha or theta bands (for full statistical details, see Table S4). Similarly, no significant relationships emerged between reaction time and post-task MST parameters, nor between reaction time and the residualized pre- to post-task changes in MST parameters, in either the eyes-closed or eyes-open condition (for full statistical details, see Tables S5 and S6).

## 4. Discussion

Recent research emphasizes the complexity of determining how brain networks support cognition and flexibly reorganize in response to changing tasks. Using EEG, we examined eyes-closed and eyes-open resting-state network integration in young adults immediately before and after a visual shape oddball task, providing insights into how baseline network states relate to performance and post-task reconfiguration.

We observed decreased global integration in the theta, lower alpha, and beta bands after task completion, independent of eye condition. This mirrors fMRI findings showing reduced post-task global efficiency in young adults [21,22] and likely reflects a transient disruption of large-scale communication, shifting toward a more modular and less demanding post-task architecture [37]. In contrast, older adults show increased post-task integration [23], suggesting age-related differences in network reorganization mechanisms.

The magnitude of post-task reduction depended on pre-task integration: participants with higher initial integration exhibited greater decreases in both eyes-closed and eyes-open conditions. This indicated that reconfiguration was proportional to the brain’s initial state, possibly reflecting a disengagement or recovery process [38,39]. Such reductions do not necessarily indicate suboptimal organization, however. While integration supports efficient global communication, it also comes with substantial wiring costs [40]. The observed pattern may reflect a shift from efficiency to economy as cognitive load diminishes, conserving energy and reallocating resources [8,40]. These dynamics demonstrate neural flexibility, highlighting the brain’s capacity to reorganize networks according to changing demands [4,41].

Eyes-open resting states consistently exhibited higher integration than eyes-closed in the upper alpha band, both before and after the task, consistent with prior MEG, EEG, and fMRI studies [25,26,27]. Discrepancies with studies reporting reduced integration [28] may reflect differences in modality, graph-theoretical approaches, or study design. The increased integration likely reflected enhanced inter-regional communication driven by sensory engagement [25] or a heightened readiness to process visual input [42].

Crucially, higher pre-task network integration in the beta frequency band during the eyes-open resting state predicted faster reaction times. To our knowledge, only one previous EEG study has linked resting-state global integration immediately before task onset with reaction time in young adults [15]. That study, focused on the eyes-closed condition, reported that shorter characteristic path length in the gamma band predicted faster responses in a Go/Nogo task. Our results extend this relationship, showing that in young adults, stronger high-frequency integration supports quicker responses independent of eye condition. This aligns with evidence from fMRI, EEG, and MEG studies linking greater resting-state network integration to higher intelligence [17], improved prospective memory performance [16], and enhanced cognitive ability [18]. Together, these results support the view that resting brain activity reflects an ongoing internal state that anticipates task demands by optimizing network configurations [43]. Greater resting-state integration may therefore reflect enhanced functional adaptability, allowing for the brain to recruit relevant brain regions to support task performance [44,45].

Task performance did not correlate with network change or post-task integration. In contrast, higher pre-task integration in the beta frequency band during the eyes-open resting-state predicted better performance and greater post-task reductions, indicating that baseline network organization, rather than immediate task outcomes, drives cognitive efficiency and the reconfiguration capacity. This highlights individual traits in intrinsic network architecture as a key determinant of neural flexibility and functional adaptability [20,46].

These findings support the concept of neural reserve, in which individual differences in network efficiency, capacity, or flexibility support task performance [47,48]. Neural reserve manifests as functional adaptability, enhancing task performance, and neural flexibility, enabling greater post-task reorganization. This interpretation is consistent with previous findings showing that young adults with higher neural reserve exhibit stronger task-related deactivations [49] and improved task performance [50]. Elevated pre-task integration thus reflects a brain that is both performance-ready and dynamically responsive—a resilient and well-resourced neural system.

Several limitations of the present study should be acknowledged. First, the analysis was restricted to frequency bands up to 20 Hz, including the lower beta range, which limits conclusions regarding higher-frequency neural activity. Second, the sample consisted exclusively of young adults, which may reduce the generalizability of the findings to older populations. Finally, the results are specific to cognitive processing in the visual modality within an oddball paradigm; network patterns may differ in auditory or multimodal tasks.

In this study, we investigated brain networks in sensor space using the phase lag index, a connectivity measure that reduces the impact of volume conduction [33]. Networks were reconstructed with MST analysis, avoiding the thresholding and normalization challenges of conventional graph approaches. Future studies should extend this framework to the source space to gain greater anatomical specificity and insight into the neural substrates of network dynamics. In addition, while the present study focused on global whole-brain metrics, examining hemispheric lateralization and the contributions of frontal, temporal, and occipital regions could yield deeper insights into the neural mechanisms underlying cognitive performance and flexibility. From an applied perspective, the proposed framework holds promise for aging and clinical research, where baseline network integration and reconfiguration capacity may serve as biomarkers of cognitive decline, neurological pathology, or compensatory plasticity. Beyond task performance, variability in alpha-band integration may also have important implications for vigilance regulation and sleep-related functioning. For example, previous research has shown that the ratio of alpha activity between pre- and post-active phases of an auditory task differs between healthy individuals and those with sleep disorders [51].

In summary, intrinsic network configuration shapes both cognitive efficiency and neural flexibility, with pre-task beta-band integration during eyes-open resting state serving as a key predictor of performance and adaptive reorganization in young adults. These findings highlight that understanding intrinsic brain network organization—including how resting-state integration varies across conditions—provides critical insight into the mechanisms by which the brain supports cognitive performance and flexibly adapts to changing task demands.

## Data Availability

The original contributions presented in the study are included in the article/Supplementary Materials; further inquiries can be directed to the corresponding author.

## Supplementary Materials

The following supporting information can be downloaded at: https://www.mdpi.com/article/10.3390/s26010041/s1.

## References

[1] Bressler S.L., Menon V. (2010). Large-scale brain networks in cognition: Emerging methods and principles. Trends Cogn. Sci..

[2] Rubinov M., Sporns O. (2010). Complex network measures of brain connectivity: Uses and interpretations. Neuroimage.

[3] Bassett D.S., Bullmore E.T., Meyer-Lindenberg A., Apud J.A., Weinberger D.R., Coppola R. (2009). Cognitive fitness of cost-efficient brain functional networks. Proc. Natl. Acad. Sci. USA.

[4] Bassett D.S., Wymbs N.F., Porter M.A., Mucha P.J., Carlson J.M., Grafton S.T. (2011). Dynamic reconfiguration of human brain networks during learning. Proc. Natl. Acad. Sci. USA.

[5] Braun U., Schäfer A., Walter H., Erk S., Romanczuk-Seiferth N., Haddad L., Schweiger J.I., Grimm O., Heinz A., Tost H. (2015). Dynamic reconfiguration of frontal brain networks during executive cognition in humans. Proc. Natl. Acad. Sci. USA.

[6] Cohen J.R., D’Esposito M. (2016). The segregation and integration of distinct brain networks and their relationship to cognition. J. Neurosci..

[7] Cole M.W., Reynolds J.R., Power J.D., Repovs G., Anticevic A., Braver T.S. (2013). Multi-task connectivity reveals flexible hubs for adaptive task control. Nat. Neurosci..

[8] Kitzbichler M.G., Henson R.N., Smith M.L., Nathan P.J., Bullmore E.T. (2011). Cognitive effort drives workspace configuration of human brain functional networks. J. Neurosci..

[9] Long Y., Liu X., Liu Z. (2023). Temporal stability of the dynamic resting-state functional brain network: Current measures, clinical research progress, and future perspectives. Brain Sci..

[10] Phan A.T., Xie W., Chapeton J.I., Inati S.K., Zaghloul K.A. (2024). Dynamic patterns of functional connectivity in the human brain underlie individual memory formation. Nat. Commun..

[11] Shi Y., Yang L., Lu J., Yan T., Ding Y., Wang B. (2024). The dynamic reconfiguration of the functional network during episodic memory task predicts the memory performance. Sci. Rep..

[12] Wang H.E., Gonzalez-Martinez J., Jirsa V., Chauvel P., Alario F.X., Liegeois-Chauvel C. (2025). Assessing functional connectivity dynamics during cognitive tasks involving the dorsal stream. Entropy.

[13] Greicius M.D., Krasnow B., Reiss A.L., Menon V. (2003). Functional connectivity in the resting brain: A network analysis of the default mode hypothesis. Proc. Natl. Acad. Sci. USA.

[14] Ramos-Loyo J., Gonzalez-Garrido A.A., Amezcua C., Guevara M.A. (2004). Relationship between resting alpha activity and the ERPs obtained during a highly demanding selective attention task. Int. J. Psychophysiol..

[15] Zhou G., Liu P., He J., Dong M., Yang X., Hou B., Von Deneen K., Qin W., Tian J. (2012). Interindividual reaction time variability is related to resting-state network topology: An electroencephalogram study. Neuroscience.

[16] Zangrossi A., Zanzotto G., Lorenzoni F., Indelicato G., Aghedu F.C., Cermelli P., Bisiacchi P.S. (2021). Resting-state functional brain connectivity predicts cognitive performance: An exploratory study on a time-based prospective memory task. Behav. Brain Res..

[17] van den Heuvel M.P., Stam C.J., Kahn R.S., Hulshoff Pol H.E. (2009). Efficiency of functional brain networks and intellectual performance. J. Neurosci..

[18] Bosma I., Reijneveld J.C., Klein M., Douw L., van Dijk B.W., Heimans J.J., Stam C.J. (2009). Disturbed functional brain networks and neurocognitive function in low-grade glioma patients: A graph theoretical analysis of resting-state MEG. Nonlinear Biomed. Phys..

[19] Kavcic V., Daugherty A.M., Giordani B. (2021). Post-task modulation of resting state EEG differentiates MCI patients from controls. Alzheimers Dement. Diagn. Assess. Dis. Monit..

[20] Martin T., Holliday E., Okhio C., Newman A., LaTella L., Mcginnis M., Požar R., Giordani B., Kavcic V. (2025). States, traits, and the resting state EEG task aftereffect. Int. J. Psychophysiol..

[21] Breckel T.P., Thiel C.M., Bullmore E.T., Zalesky A., Patel A.X., Giessing C. (2013). Long-term effects of attentional performance on functional brain network topology. PLoS ONE.

[22] Lin P., Yang Y., Gao J., De Pisapia N., Ge S., Wang X., Zuo C.S., Levitt J.J., Niu C. (2017). Dynamic default mode network across different brain states. Sci. Rep..

[23] Požar R., Kero K., Martin T., Giordani B., Kavcic V. (2023). Task aftereffect reorganization of resting state functional brain networks in healthy aging and mild cognitive impairment. Front. Aging Neurosci..

[24] Weng Y., Liu X., Hu H., Huang H., Zheng S., Chen Q., Song J., Cao B., Wang J., Wang S. (2020). Open eyes and closed eyes elicit different temporal properties of brain functional networks. Neuroimage.

[25] Jin S.H., Jeong W., Lee D.S., Jeon B.S., Chung C.K. (2014). Preserved high-centrality hubs but efficient network reorganization during eyes-open state compared with eyes-closed resting state: An MEG study. J. Neurophysiol..

[26] Tan B., Kong X., Yang P., Jin Z., Li L. (2013). The difference of brain functional connectivity between eyes-closed and eyes-open using graph theoretical analysis. Comput. Math. Methods Med..

[27] Wang X.H., Li L., Xu T., Ding Z. (2015). Investigating the temporal patterns within and between intrinsic connectivity networks under eyes-open and eyes-closed resting states: A dynamical functional connectivity study based on phase synchronization. PLoS ONE.

[28] Xu P., Huang R., Wang J., Van Dam N.T., Xie T., Dong Z., Chen C., Gu R., Zang Y.-F., He Y. (2014). Different topological organization of human brain functional networks with eyes open versus eyes closed. Neuroimage.

[29] Stam C.J., Tewarie P., van Dellen E., van Straaten E.C., Hillebrand A., Van Mieghem P. (2014). The trees and the forest: Characterization of complex brain networks with minimum spanning trees. Int. J. Psychophysiol..

[30] Green D.M., Swets J.A. (1966). Signal Detection Theory and Psychophysicsi.

[31] Delorme A., Makeig S. (2004). EEGLAB: An open-source toolbox for analysis of single-trial EEG dynamics. J. Neurosci. Methods.

[32] Makeig S., Debener S., Onton J., Delorme A. (2004). Mining event-related brain dynamics. Trends Cogn. Sci..

[33] Stam C.J., Nolte G., Daffertshofer A. (2007). Phase lag index: Assessment of functional connectivity from multi channel EEG and MEG with diminished bias from common sources. Hum. Brain Mapp..

[34] Kruskal J.B. (1956). On the shortest spanning subtree of a graph and the traveling salesman problem. Proc. Am. Math. Soc..

[35] Hagberg A.A., Schult D.A., Swart P.J. (2008). Exploring Network Structure, Dynamics, and Function Using NetworkX.

[36] Benjamini Y., Hochberg Y. (1995). Controlling the false discovery rate: A practical and powerful approach to multiple testing. J. R. Stat. Soc. Ser. B Methodol..

[37] Meunier D., Lambiotte R., Bullmore E.T. (2010). Modular and hierarchically modular organization of brain networks. Front. Neurosci..

[38] Daselaar S.M., Prince S.E., Cabeza R. (2004). When less means more: Deactivations during encoding that predict subsequent memory. Neuroimage.

[39] Wang Z., Liu J., Zhong N., Qin Y., Zhou H., Li K. (2012). Changes in the brain intrinsic organization in both on-task state and post-task resting state. Neuroimage.

[40] Bullmore E., Sporns O. (2012). The economy of brain network organization. Nat. Rev. Neurosci..

[41] Yin W., Li T., Hung S.-C., Zhang H., Wang L., Shen D., Zhu H., Mucha P.J., Cohen J.R., Lin W. (2020). The emergence of a functionally flexible brain during early infancy. Proc. Natl. Acad. Sci. USA.

[42] Costumero V., Bueichekú E., Adrián-Ventura J., Ávila C. (2020). Opening or closing eyes at rest modulates the functional connectivity of V1 with default and salience networks. Sci. Rep..

[43] Deco G., Jirsa V.K., McIntosh A.R. (2011). Emerging concepts for the dynamical organization of resting-state activity in the brain. Nat. Rev. Neurosci..

[44] Allen E.A., Damaraju E., Plis S.M., Erhardt E.B., Eichele T., Calhoun V.D. (2014). Tracking whole-brain connectivity dynamics in the resting state. Cereb. Cortex.

[45] Shine J.M., Bissett P.G., Bell P.T., Koyejo O., Balsters J.H., Gorgolewski K.J., Moodie C.A., Poldrack R.A. (2016). The dynamics of functional brain networks: Integrated network states during cognitive task performance. Neuron.

[46] Harmelech T., Malach R. (2013). Neurocognitive biases and the patterns of spontaneous correlations in the human cortex. Trends Cogn. Sci..

[47] Stern Y. (2002). What is cognitive reserve? Theory and research application of the reserve concept. J. Int. Neuropsychol. Soc..

[48] Stern Y. (2009). Cognitive reserve. Neuropsychologia.

[49] Stern Y., Zarahn E., Habeck C., Holtzer R., Rakitin B.C., Kumar A., Flynn J., Steffener J., Brown T. (2008). A common neural network for cognitive reserve in verbal and object working memory in young but not old. Cereb. Cortex.

[50] Speer M.E., Soldan A. (2015). Cognitive reserve modulates ERPs associated with verbal working memory in healthy younger and older adults. Neurobiol. Aging.

[51] Runnova A., Selskii A., Kisele A., Shamionov R., Parsamyan R., Zhuravlev M. (2021). Changes in EEG Alpha Activity during Attention Control in Patients: Association with Sleep Disorders. J. Pers. Med..

